# Optimization of a 3D Skin Equivalent Incorporating Melanocytes or Melanoma Cells for In Vitro Melanoma Research

**DOI:** 10.3390/bioengineering13090980

**Published:** 2026-08-26

**Authors:** Sylwia Hasterok, Skaidre Jankovskaja, Zdenka Prgomet, Lars Ohlsson, Anna Gustafsson

**Affiliations:** 1 Department of Biomedical Science, Faculty of Health and Society, Malmö University, 214 28 Malmö, Sweden; skaidre.jankovskaja@mau.se (S.J.); zdenka.prgomet@mau.se (Z.P.); lars.ohlsson@mau.se (L.O.); 2 Biofilms Research Center for Biointerfaces, Malmö University, 214 28 Malmö, Sweden; 3 Section for Oral Biology and Pathology, Faculty of Odontology, Malmö University, 214 21 Malmö, Sweden

**Keywords:** 3D cell culture, in vitro model, methodological study, melanoma, kynurenine pathway, UVB

## Abstract

Replicating the complex physiology of human skin in vitro remains a challenge for cutaneous oncology. Although several three-dimensional (3D) skin and melanoma models have been developed, reproducible skin equivalent systems that enable direct and standardized comparisons between healthy and melanoma-containing skin remain valuable experimental tools. This study aimed to optimize and characterize skin equivalents containing either melanocytes or melanoma cells and to evaluate their utility as platforms for investigating melanoma-associated skin biology and UVB-induced responses in vitro. To achieve this, 3D skin equivalents containing fibroblasts, keratinocytes, and either melanocytes or melanoma cells were reconstructed on a polystyrene scaffold to generate healthy (mc) and melanoma (mm) models. Morphological characteristics were compared with clinically verified human tissue sections, and functional responses to UVB irradiation were assessed with a focus on the kynurenine pathway. The models demonstrated a distinct dermal–epidermal architecture and distinguishable melanoma-associated features, including epidermis-confined melanoma cell clusters, in mm constructs. UVB exposure induced differential responses between models, including significant differences in kynurenine pathway regulation. These findings suggest that the developed skin equivalents provide a reproducible in vitro platform for studying melanoma-associated skin biology and treatment-related metabolic responses and may support future mechanistic studies of skin cancer progression.

## 1. Introduction

The skin, the body’s largest organ, functions as both a sensory structure and a protective barrier between the body and its surroundings [1]. A complex and dynamic tissue, the skin is crucial for maintaining homeostasis through regulation of biological processes essential to human health. Structurally, the skin is composed of two principal layers, the epidermis and the underlying dermis, which together contribute to barrier function and tissue homeostasis [1]. Among the specialized cell populations residing in the epidermis are melanocytes, which are responsible for melanin production and protection against ultraviolet (UV) radiation [2]. These cells can undergo malignant transformation, giving rise to melanoma. UV radiation is classified as human carcinogen that contributes to skin inflammation, aging, and cancer through both direct and indirect mechanisms. There are three types of UV light, out of which ultraviolet B (UVB) is the primary driver of melanoma initiation and development through its direct mutagenic effects on DNA [3]. In addition to inducing DNA damage, UVB exposure alters the skin microenvironment through inflammatory and metabolic responses that may contribute to melanoma progression [4].

While animal models have contributed substantially to our understanding of melanoma biology, important interspecies differences limit their ability to fully recapitulate human skin biology [5]. Differences in skin thickness, permeability, melanocyte distribution, and melanoma origin between commonly used animal models and humans may affect the translational relevance of experimental findings [6,7]. In addition, ethical considerations and the increasing emphasis on 3Rs principles (Replacement, Reduction, and Refinement) have further driven the development of physiologically relevant human in vitro models [8].

Three-dimensional (3D) skin equivalents have emerged as valuable tools for studying melanoma biology because they more closely reproduce the structural organization and cellular interactions of human skin than conventional monolayer cultures. Current models range from melanoma spheroids and organoids to full-thickness skin equivalents incorporating keratinocytes, fibroblasts, melanocytes, and melanoma cells [9,10,11,12,13]. These systems have provided important insights into tumor–microenvironment interactions, melanoma invasion, and therapeutic responses while reducing reliance on animal models. Full-thickness skin equivalents typically comprise a stratified epidermal compartment supported by a fibroblast-populated dermal matrix and can be adapted to model different stages of melanoma progression through the incorporation of melanoma cells within the reconstructed tissue [14].

Despite these advances, variability in cellular composition, scaffold materials, and experimental design can complicate direct comparisons between studies. Consequently, there remains a need for standardized and well-characterized reconstructed skin equivalents that enable direct comparison between healthy melanocyte-containing and melanoma-containing skin under identical experimental conditions. Such side-by-side systems provide a physiologically relevant platform for investigating how alterations in tissue architecture and cellular interactions influence melanoma-associated biological processes and responses to environmental factors such as UV radiation.

Beyond its well-established role in inducing DNA damage and mutagenesis, UV radiation influences several metabolic and immunoregulatory pathways within the skin microenvironment. These responses contribute to the regulation of skin homeostasis and may affect melanoma initiation and progression [15]. One pathway that has attracted increasing interest in cancer biology is the kynurenine pathway, the major metabolic route for tryptophan (Trp) catabolism, leading to the formation of kynurenine (Kyn) and contributing to nicotinamide adenine dinucleotide (NAD+) biosynthesis, an essential cofactor in numerous cellular processes [16]. Indoleamine 2,3-dioxygenase (IDO-1) is one of the three key rate-limiting enzymes regulating Trp catabolism into Kyn under physiological conditions. As an inducible enzyme, IDO-1 requires external stimuli for activation, with interferon-gamma (IFN-γ) serving as its primary inducer. Another mechanism for IDO-1 upregulation involves a positive feedback loop mediated by the pro-inflammatory cytokine interleukin-6 (IL-6) [17]. IDO-1 is known to be upregulated in several cancer types, including melanoma, underscoring its potential significance in cancer pathology [18]. Furthermore, dysregulation of Trp metabolism through the Kyn pathway has been associated with immune evasion in melanoma, thereby promoting tumor progression [19].

The aim of this study was to optimize and characterize 3D skin equivalents containing either melanocytes or melanoma cells and to evaluate their morphological resemblance to human skin. In addition, the utility of the models as experimental platforms was assessed by examining UVB-induced responses associated with kynurenine pathway regulation.

## 2. Materials and Methods

### 2.1. 3D Skin Equivalent Reconstruction Protocol

Both 3D skin equivalents were prepared in-house using a modified protocol outlined by Zoio et al. [20]. The reconstructed models consisted of a melanocyte-containing model (mc model) and a melanoma-containing model (mm model). Modifications to the original protocol included changes in media composition, fibroblast type, timing of certain formation stages, and melanoma cell integration. The composition of cell culture media is described in Table 1.

#### 2.1.1. Fibroblast Cell Culture (Week −1)

Neonatal Dermal Primary Fibroblast cell line, BJ (ATCC^®^; CRL-2522; Manassas, VA, USA) was thawed one week prior (week −1) to the initiation of the 3D skin equivalent assembly and was cultured in Eagle’s Minimum Essential Medium (EMEM; ATCC, Manassas, VA, USA) supplemented with 10% heat-inactivated fetal bovine serum (FBS; Thermo Fisher Scientific, Waltham, MA, USA) and 1% penicillin/streptomycin (PEST; Gibco, Waltham, MA, USA). The fibroblasts were thawed at passage 2 and expanded in monoculture by being split twice over the course of one week.

#### 2.1.2. Initiation of the 3D Skin Equivalent Assembly (Week 0)

The 3D skin equivalents were reconstructed using porous polystyrene scaffolds (Alvetex^TM^ 12 Well Inserts; Reprocell, Beltsville, MD, USA) selected for their ~200 µm thickness and high porosity (~90%), which support fibroblast infiltration. Scaffolds were rendered hydrophilic per the manufacturer’s protocol, transferred to 12-well plates (VWR International, Radnor, PA, USA), and washed twice with 1 mL of the fibroblast culture medium per insert. Inserts were then distributed across two new 12-well plates (6 inserts each).

Human fibroblasts were detached using Accutase (Gibco, Waltham, MA, USA), counted manually, and seeded onto each scaffold at 1.0 × 10^6^ cells in 100 µL medium. The plates were incubated for 2 h (37 °C; 5% CO_2_) to promote cell attachment. After incubation, 1.5 mL of EMEM containing 10% FBS, 1% PEST, freshly supplemented with 100 µg/mL L-ascorbic acid 2-phosphate (Sigma-Aldrich, Burlington, MA, USA) was added to the bottom of each insert, while 500 µL was added to the top. Empty wells were filled with PBS to maintain humidity. Constructs were cultured for 14 days (37 °C; 5% CO_2_) to allow fibroblast proliferation and extracellular matrix (ECM) deposition. Medium was replaced three times per week by gently rotating inserts upside-down to discard the old medium, transferring them to new plates, and replenishing with fresh medium.

#### 2.1.3. Melanoma Cells and Melanocyte Cell Culture (Week +1)

The Adult Primary Epidermal Melanocytes, HEMa (ATCC^®^; PCS-200-013; Manassas, VA, USA) and the Melanoma Cell Line, Mel-HO (German Collection of Microorganisms and Cell Cultures, DSMZ; ACC 62; Braunschweig, Germany) were thawed one week after (week +1) fibroblast incorporation into the polystyrene scaffolds was established (week 0). Melanocytes were cultured in Dermal Cell Basal Medium (ATCC) supplemented with the Adult Melanocyte Growth Kit (ATCC), and 1% PEST. Melanoma cells were cultured in Dulbecco’s Modified Eagle Medium (DMEM) with 1 g/L glucose (Gibco) supplemented with 10% FBS (Thermo Fisher Scientific, Waltham, MA, USA) and 1% PEST. HEMa were thawed at passage 2, while Mel-HO at passage 19. Both cell types were expanded in monocultures by being split twice over the course of one week.

#### 2.1.4. Keratinocyte Cell Culture (Week +1 + 24 h)

Neonatal Primary Human Epidermal Keratinocytes, HEKn (derived from neonatal foreskin, ATCC^®^; PCS-200-010; Manassas, VA, USA) were thawed 24 h after melanocytes and melanoma cells (week +1 + 24 h) and cultured in EpiLife Medium with 60 µM calcium (Thermo Fisher Scientific, Waltham, MA, USA) supplemented with Human Keratinocyte Growth Supplement (HKGS; final concentration: 1%; Gibco, Waltham, MA, USA) and gentamicin sulfate (final concentration: 0.2%; Corning, Corning, NY, USA). The keratinocytes were thawed at passage 2 and expanded in monoculture by being split twice over the course of one week.

#### 2.1.5. Melanoma Cells or Primary Melanocyte Incorporation into 3D Skin Equivalents (Week +2) 

Depending on the model, melanoma cells (Mel-HO) or primary melanocytes (HEMa) were detached using Accutase and counted manually. The medium from the top of the scaffolds was removed, the inserts were transferred to new plates, and 5.0 × 10^4^ Mel-HO or HEMa in 100 µL of medium each were seeded onto the fibroblast-enriched scaffolds. The number of cells was chosen based on the physiological melanocyte-to-keratinocyte ratio of 1:10 found in the basal layer of native human epidermis [21]. The plates were incubated for 2 h (37 °C; 5% CO_2_) to ensure cell adhesion. After incubation, 1.5 mL of fibroblast culture medium freshly supplemented with 100 µg/mL L-ascorbic acid 2-phosphate was added to the bottom of the wells, and 500 µL of melanocyte or melanoma culture medium to the top of the inserts, followed by a 24 h incubation (37 °C; 5% CO_2_).

#### 2.1.6. Primary Keratinocyte Incorporation into 3D Skin Equivalents (Week +2 + 24 h)

After 24 h, the primary keratinocytes (HEKn) were detached with Accutase and counted manually. The culture medium was removed from the top of the inserts, and the inserts were placed in new 12-well plates. Next, 5.0 × 10^5^ HEKn in 100 µL of medium were seeded onto each model and incubated for 2 h (37 °C; 5% CO_2_) to allow cell adhesion. Following incubation, the bottom of the wells (1.5 mL) and the top of the inserts (500 µL) were refilled with mm or mc model co-culture growth medium as described in Table 2. Both constructs were submerged for 3 days (37 °C; 5% CO_2_), with the cell culture media changed once during this period.

#### 2.1.7. Keratinocyte Differentiation at Air-Liquid Interface (Week +2 + 96 h)

**Figure 1:**
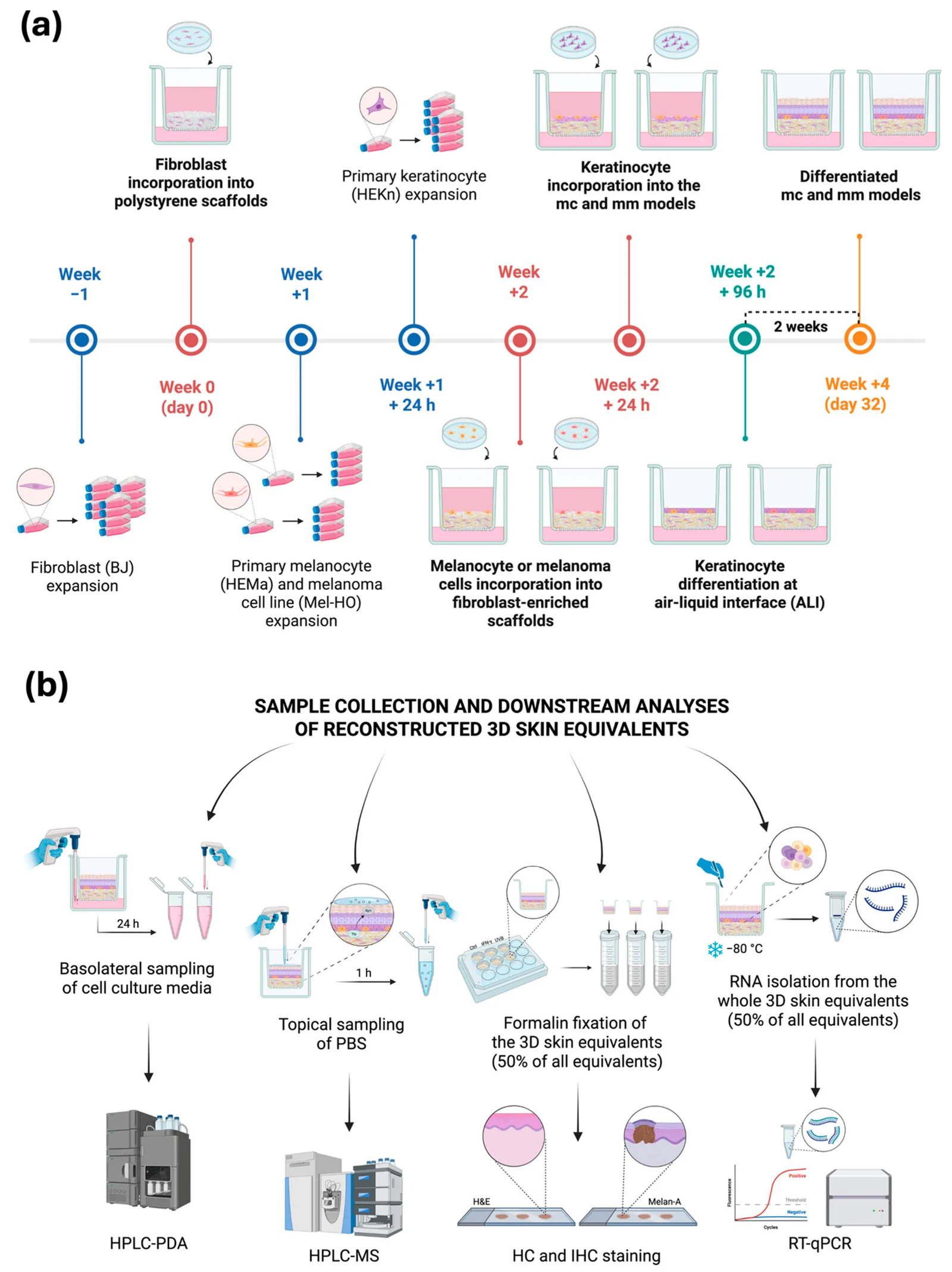
Timeline of mc and mm model reconstruction process (**a**) and sampling collection procedures with downstream analyses (**b**). (**a**) Blue timepoints indicate the thawing and expansion of specific primary cells or cell lines. Red timepoints mark the incorporation of cells into mc and mm models. The green time point represents the initiation of the air-liquid interface (ALI), while the orange time point signifies the completion of mc and mm model differentiation. The entire reconstruction process from the start (day 0) to completion spans 32 days. (**b**) Sampling procedures include (left) collection of cell culture media (basolateral) or PBS (topical) for HPLC-PDA or HPLC-MS analysis, and (right) formalin fixation or RNA isolation from both 3D skin equivalents, followed by HC and IHC staining or RT-qPCR. Topical and basolateral samples (left) were collected in duplicate for each condition. For each model type (mc and mm), one membrane per condition was used for RNA isolation and the other for formalin fixation (right). HPLC-PDA, high-performance liquid chromatography with a photodiode array; HPLC-MS, high-performance liquid chromatography-mass spectrometry; HC, histochemical staining; IHC, immunohistochemical staining; RT-qPCR, reverse transcriptase quantitative polymerase chain reaction. Created with BioRender.com.

After three days, the constructs were transferred to an air-liquid interface (ALI) to promote keratinocyte differentiation into distinct layers. This process involved carefully removing all co-culture medium from the top of the inserts and transferring the models to new 12-well plates, each containing 1.5 mL of the appropriate mc or mm differentiation medium as described in Table 3. Keratinocyte differentiation lasted 14 days (37 °C; 5% CO_2_), during which the differentiation media were changed three times per week. A timeline of the mc and mm model reconstruction process is presented in Figure 1a.

### 2.2. Stimulation, Sampling Collection Procedures & Downstream Analyses

Once the differentiated mc and mm models were developed, they were divided into three groups each: non-exposed models (Control; Ctrl), IFN-γ-stimulated models (20 ng/mL; R&D Systems, Minneapolis, MN, USA), and UVB-exposed models (80 mJ/cm^2^). 

In total, 36 skin equivalents were reconstructed, comprising 18 mc models and 18 mm models. These were generated across three independent experimental repeats using the same cell types. Each experimental repeat included two technical replicates per condition (Ctrl, IFN-γ, UVB). While this design does not capture biological variability between donors, it ensures reproducibility of the model.

Prior to stimulation, the culture medium in the lower (basolateral) compartment of all inserts was removed and replaced with a fresh differentiation medium. IFN-γ stimulation was performed by supplementing the medium with 20 ng/mL of IFN-γ, whereas UVB exposure was applied using a UV lamp (model UVM-57; UVP 95-0104-02; l = 302 nm; Analytik Jena, Jena, Germany) at a dose of 80 mJ/cm^2^. All 3D skin equivalents were subsequently incubated for 24 h prior to sample collection.

Following stimulation, 2 × 700 µL of the cell culture media constituting the basolateral samples of mc and mm models were collected from the bottom of each insert, transferred into individual Eppendorf tubes, and stored at −80 °C until analysis using High-Performance Liquid Chromatography with a photodiode array (HPLC-PDA). 

For topical sampling, 500 µL of PBS was applied to the surface of each mc and mm model, followed by 1 h incubation (37 °C; 5% CO_2_). Approximately 500 µL of PBS from each mc and mm model was then collected into individual Eppendorf tubes and stored at −80 °C until analysis using High-Performance Liquid Chromatography–Mass Spectrometry (HPLC-MS). 

Before histochemical (HC) and immunohistochemical (IHC) staining, one technical replicate of each mc and mm model per testing condition (50% of all 3D skin equivalents) was fixed in a 4% formaldehyde solution. This process involved transferring the entire inserts, each containing a complete 3D skin equivalent, into individual 50 mL tubes filled with formaldehyde solution and keeping them at room temperature (RT) for 24 h until HC and IHC staining were performed.

Subsequently, the scaffolds with the remaining half of the mc and mm models of each type were carefully cut out of the inserts, placed into individual 1.5 mL Eppendorf tubes, and stored at −80 °C until further analysis with Reverse Transcriptase Quantitative Polymerase Chain Reaction (RT-qPCR). All sampling collection procedures and downstream analyses are summarized in Figure 1b, with downstream analyses described in detail in Appendix A.

### 2.3. Statistical Analysis

All measurement data are presented as mean ± standard error of the mean (SEM), based on three independent 3D skin equivalent reconstructions (unless otherwise noted in figure legends). Results are shown as normalized values, expressed as the ratio of stimulated to unstimulated samples. For HPLC-PDA results, peak area integration was performed automatically using OpenLAB CDS ChemStation (LC and CE Drivers, version A.02.10; Intelligent Reporting, version A.01.08.108; Agilent Technologies; Santa Clara, CA, USA) followed by manual peak inspection. Analysis of HPLC-MS data was conducted using MassLynx V4.1 software (Waters; Milford, MA, USA). The statistical significance of differences was evaluated using the paired samples *t*-test. A *p*-value of <0.05 was considered statistically significant (* *p* ≤ 0.05, ** *p* ≤ 0.01). Analyses were performed with GraphPad Prism, version 10.1.0 (GraphPad Inc.; San Diego, CA, USA).

## 3. Results

### 3.1. The Melanocyte Model Shows Similarities to the Morphology of Normal Human Skin

To assess whether the 3D culture of fibroblasts, melanocytes, and keratinocytes replicated the key morphological features of normal skin, both the mc model and native tissue were stained with hematoxylin and eosin (H&E). 

**Figure 2:**
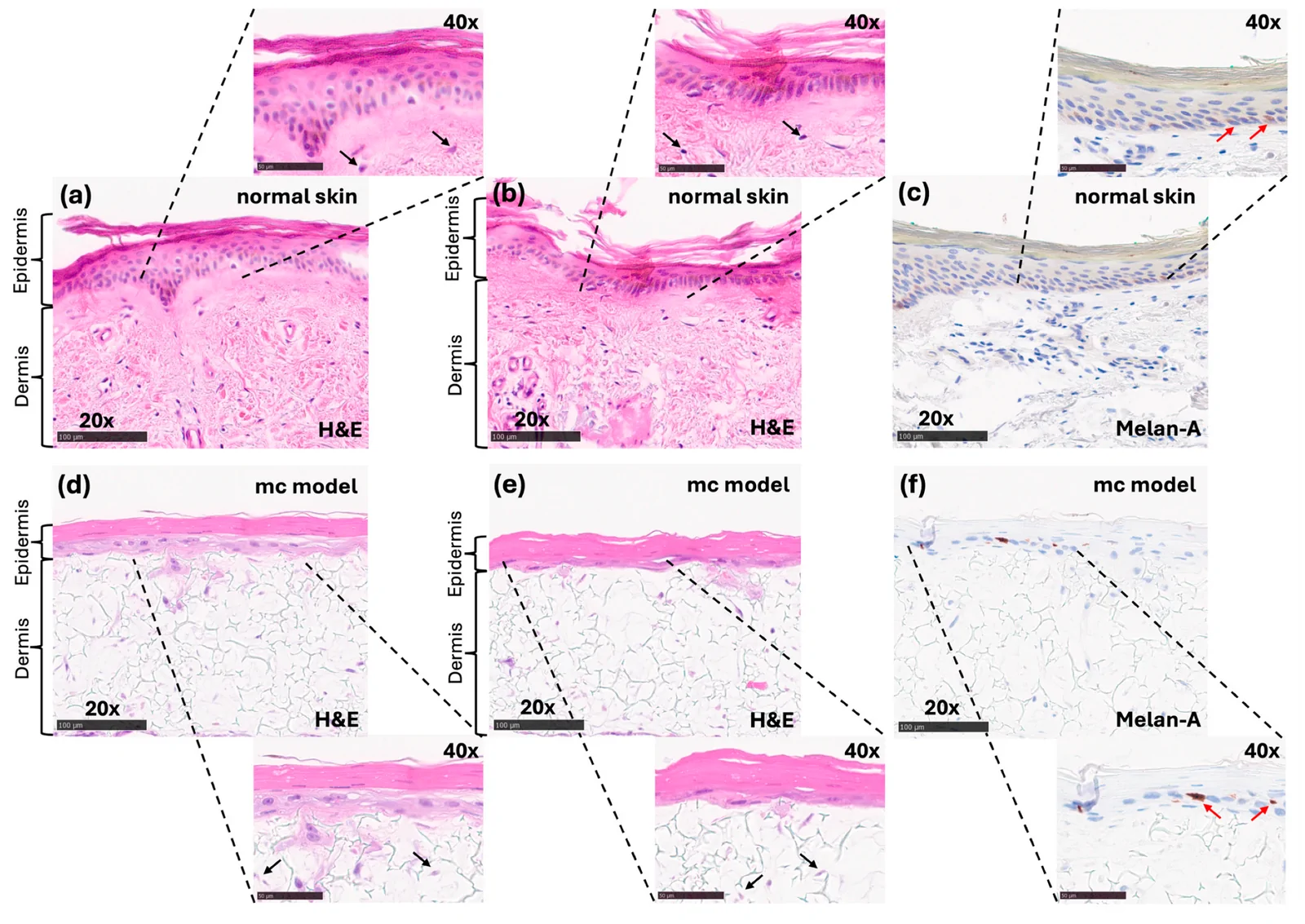
Histochemical and immunohistochemical comparison of normal human skin and mc model. Morphology of normal human skin (**a**,**b**) and the 3D cell culture with melanocytes (mc model: (**d**,**e**)) stained with H&E. Identification of melanocytes in normal human skin (**c**) and the mc model (**f**) using Melan-A staining. Representative images from one tissue section each of normal skin (**a**,**b**) and three independent experiments of the mc model (**d**,**e**) are shown. Black arrows indicate the dermal location of fibroblasts, while red arrows indicate the location of melanocytes. H&E, hematoxylin and eosin; Melan-A, melanoma antigen recognized by T cells. Scale bars correspond to 100 µm in panels acquired 20× magnification and 50 µm in panels acquired 40× magnification.

Histological analysis of native human skin (Figure 2a,b) demonstrated the expected epidermal and dermal architecture, including a well-stratified epidermis with distinct epidermal layers and a fibroblast-containing dermis. Melanocytes’ presence was confirmed via immunostaining for Melan-A (Figure 2c). 

**Figure A1:**
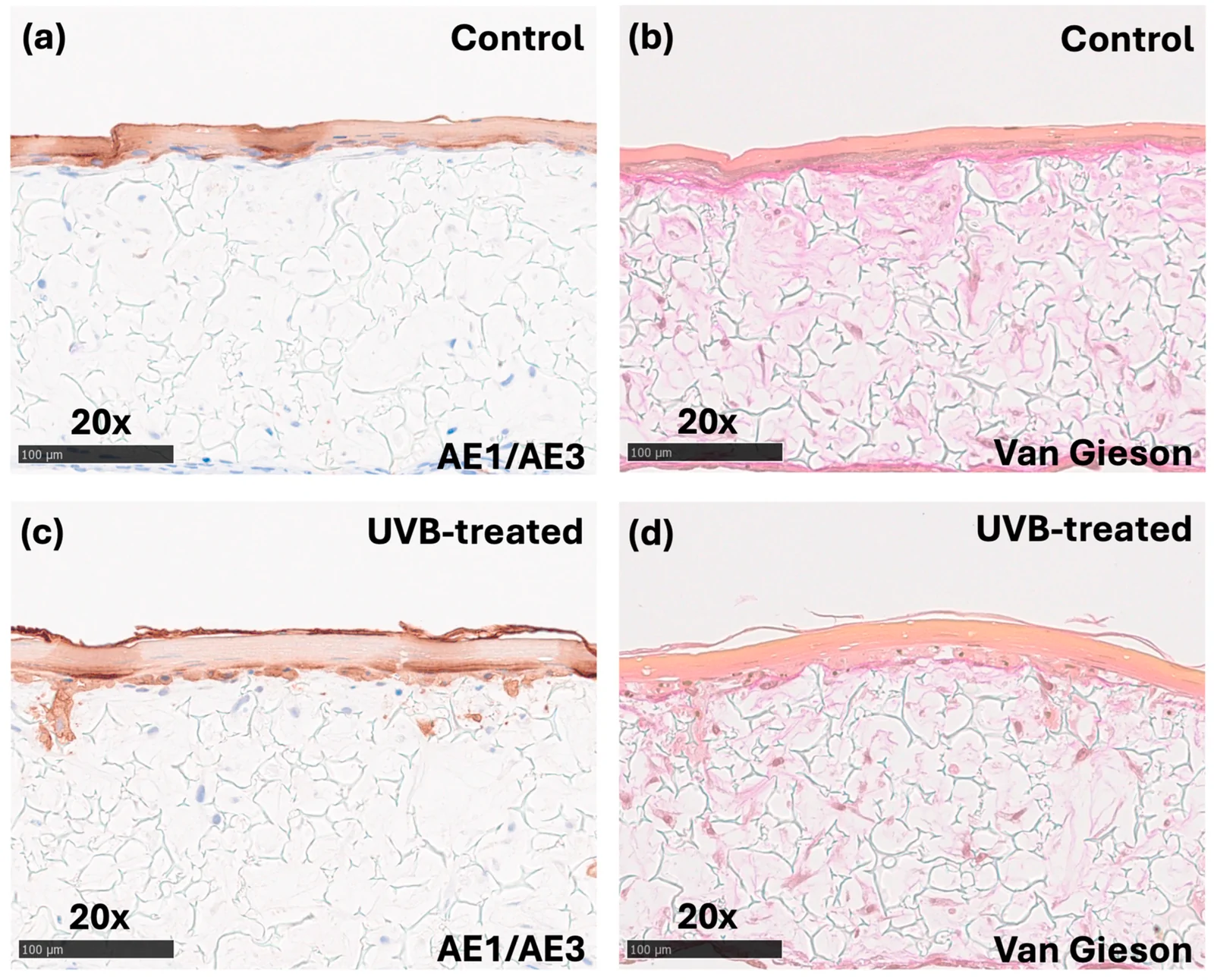
Histological and immunohistochemical characterization of the reconstructed mc model. Representative AE1/AE3 immunohistochemical staining of the reconstructed mc model under control (**a**) and UVB-treated (**c**) conditions, demonstrating the reconstructed epidermal compartment with brown DAB chromogenic staining. Representative Van Gieson staining of the reconstructed mc model under control (**b**) and UVB-treated (**d**) conditions, demonstrating the reconstructed dermal compartment with collagen fibers stained pink. Scale bars = 100 µm.

The mc model (Figure 2d,e) formed epidermis-like and dermal compartments. Fibroblasts were distributed within the scaffold-based dermal region, while Melan-A-positive melanocytes were detected predominantly in the basal region of the epidermal-like layer (Figure 2f). Compared with native human skin, the epidermal compartment of the mc model was thinner and displayed limited stratification, with regions containing only a few keratinocyte layers. A stratum-corneum-like surface layer was present, although its morphology differed from that observed in native skin. In some regions, the cornified layer appeared more compact than in native human skin, and occasional focal separation within the epidermal compartment was observed. Variability in epidermal thickness was observed throughout the construct. In some regions, downward extensions of epidermal cells into the scaffold were present (Figure 2d). Additional Van Gieson staining demonstrated collagen deposition within the reconstructed dermal compartment, while AE1/AE3 immunohistochemistry confirmed the reconstructed epidermal compartment in both control and UVB-treated mc models (Appendix B, Figure A1).

### 3.2. The 3D Culture with Melanoma Cells as an In Vitro Model of Non-Healthy Skin

**Figure 3:**
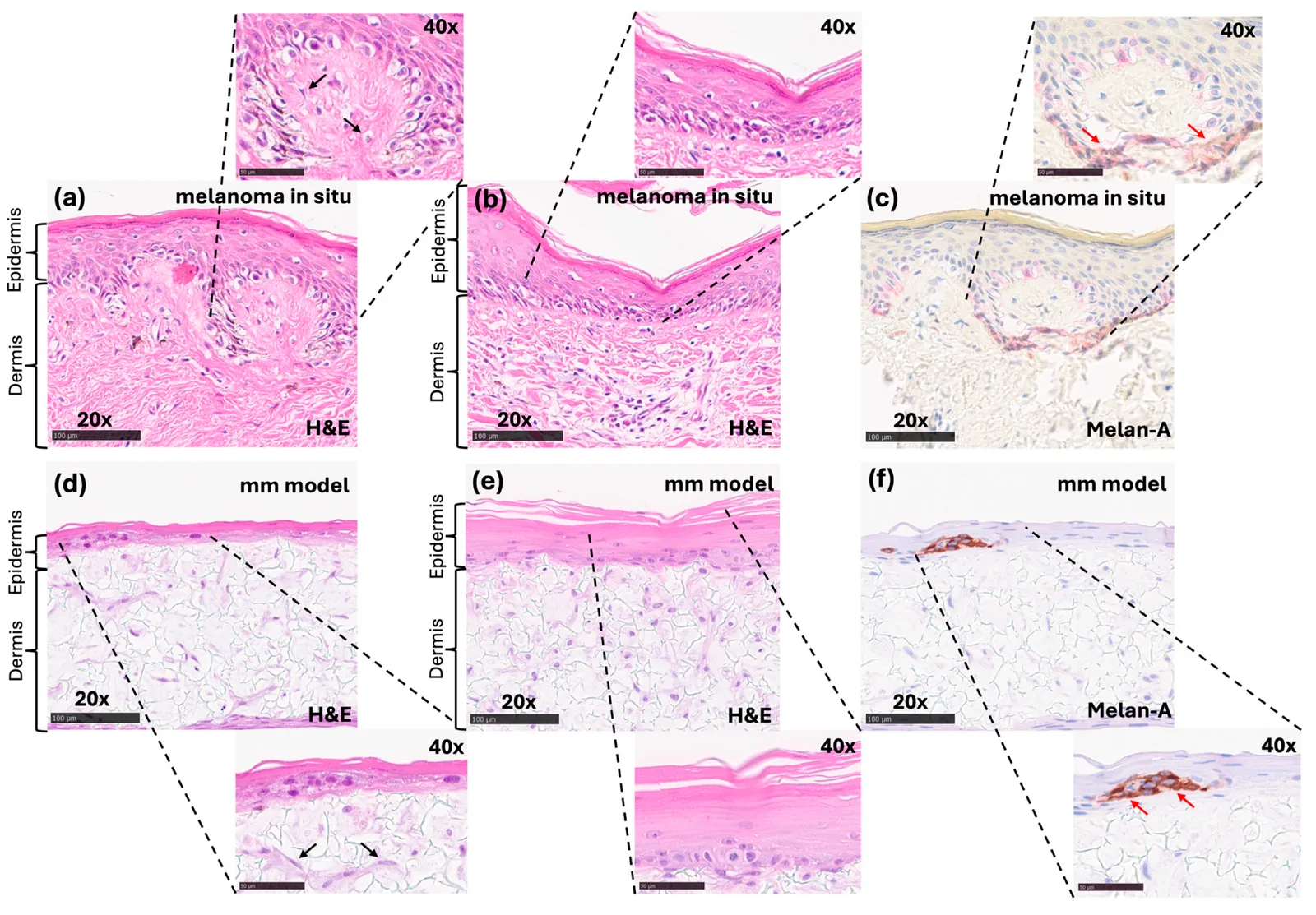
Histochemical and immunohistochemical comparison of human skin with melanoma in situ and mm model. Panels (**a**,**b**) and (**d**,**e**) show H&E-stained sections of melanoma in situ and the mm model, respectively. Panels (**c**,**f**) highlight melanoma cells identified using the Melan-A antibody in both melanoma in situ and the mm model. Representative images from one tissue section each of melanoma in situ (**a**,**b**) and three independent experiments of the mm model (**d**,**e**) are shown. Black arrows indicate the dermal location of fibroblasts, while red arrows indicate the location of melanoma cells. H&E, hematoxylin and eosin; Melan-A, melanoma antigen recognized by T cells. Scale bars correspond to 100 µm in panels acquired 20× magnification and 50 µm in panels acquired 40× magnification.

To evaluate the 3D culture as an in vitro melanoma model, melanoma cells were incorporated into the construct and analyzed using H&E and Melan-A staining. Native melanoma in situ tissue displayed the expected histological architecture, including a cellular dermis, a stratified epidermis, and melanoma cell nests within the epidermal compartment (Figure 3a–c).

The mm model (Figure 3d–f) formed distinct epidermal-like and dermal compartments containing melanoma cell clusters within the epidermal region. The dermal compartment contained fibroblasts distributed throughout the scaffold structure, while the epidermal-like layer remained thinner and less organized compared with native melanoma tissue. In regions surrounding melanoma cell clusters, the viable epidermal-like layer appeared reduced in thickness (Figure 3d). 

**Figure A2:**
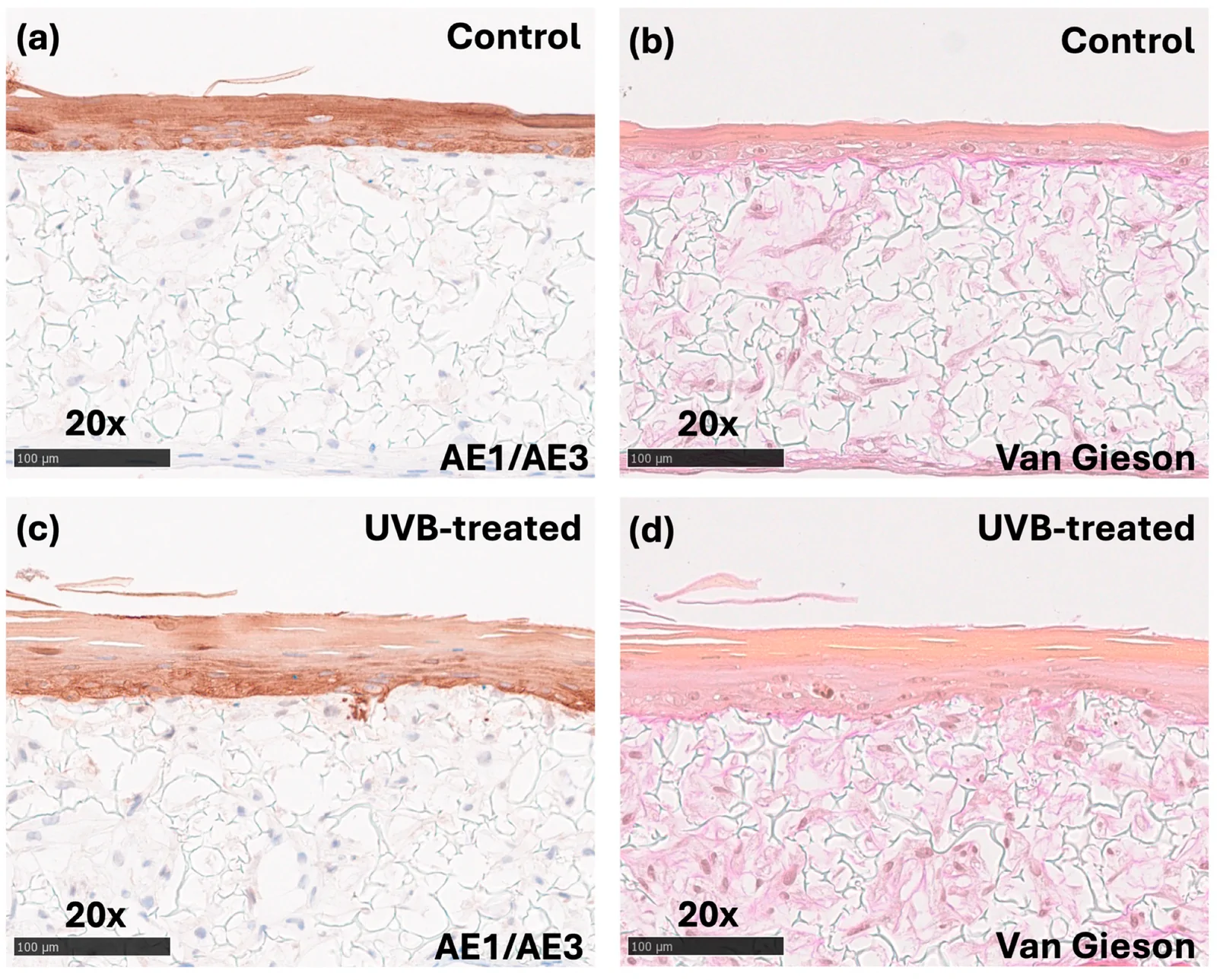
Histological and immunohistochemical characterization of the reconstructed mm model. Representative AE1/AE3 immunohistochemical staining of the reconstructed mm model under control (**a**) and UVB-treated (**c**) conditions, demonstrating the reconstructed epidermal compartment with brown DAB chromogenic staining. Representative Van Gieson staining of the reconstructed mm model under control (**b**) and UVB-treated (**d**) conditions, demonstrating the reconstructed dermal compartment with collagen fibers stained pink. Scale bars = 100 µm.

Melan-A staining confirmed the presence of melanocytic cells within cell clusters in the mm model (Figure 3f). These clusters were primarily localized within the epidermal-like compartment and exhibited heterogeneous size and distribution patterns. The observed organization was consistent with a radial growth phase-like pattern, with cells confined to the epidermal compartment in the analyzed sections. Similar to the mc model, Van Gieson staining demonstrated collagen deposition within the reconstructed dermal compartment, while AE1/AE3 immunohistochemical staining confirmed the presence of the reconstructed epidermal compartment in both control and UVB-treated models (Appendix B, Figure A2).

### 3.3. Stronger IL-6 and IDO-1 Response to UVB in the Melanoma Model

To demonstrate the utility of the mc and mm models as functional tools, both were investigated with respect to the Kyn pathway in which IL-6 and IDO-1 play significant regulatory roles.

**Figure 4:**
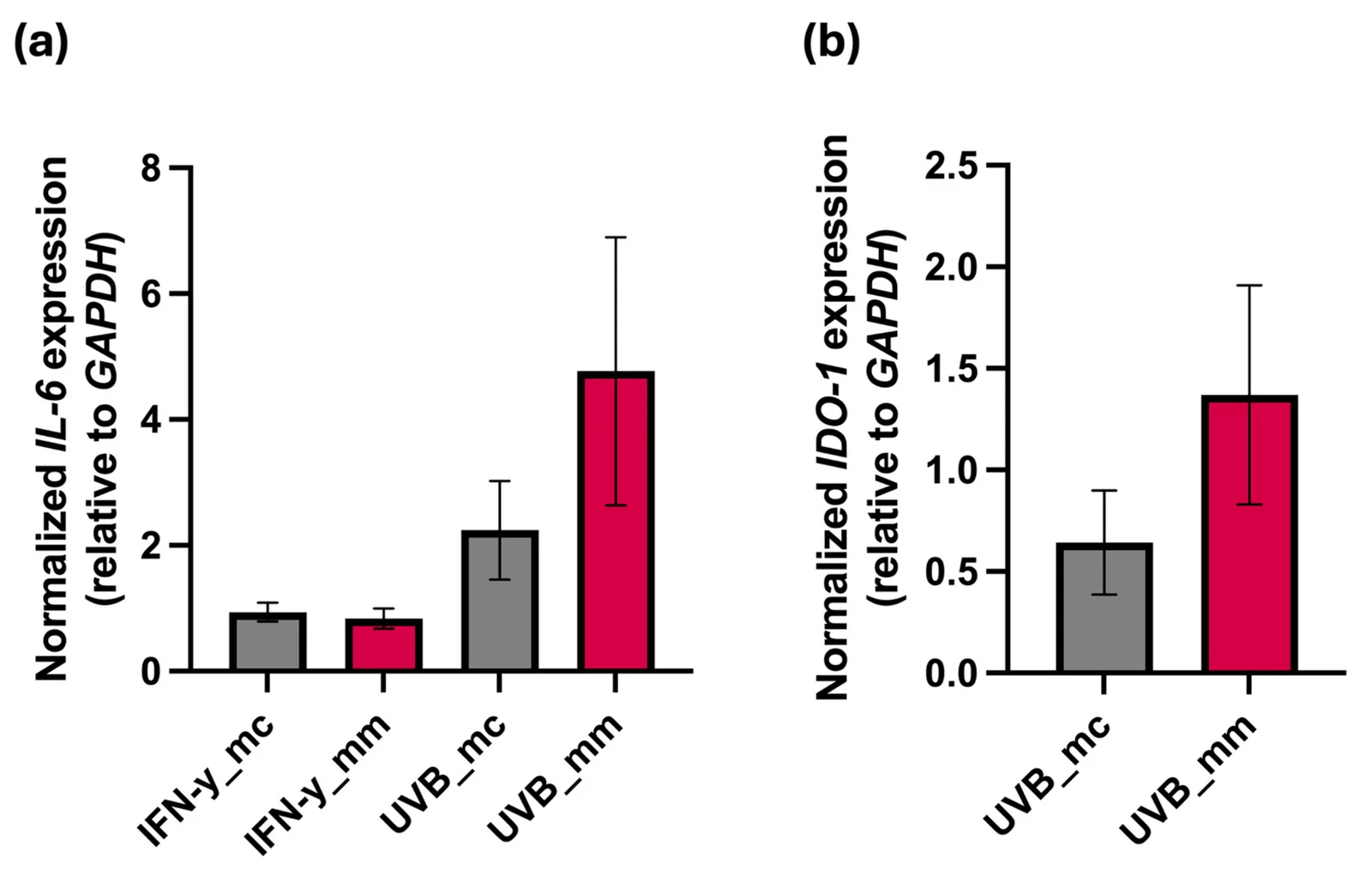
Impact of IFN-γ stimulation or UVB exposure on IL-6 expression (**a**), and the effect of UVB exposure on IDO-1 expression (**b**) in mc and mm models. Gray bars represent the mc model, and red bars represent the mm model. RNA was extracted from the models following treatments with IFN-γ (20 ng/mL) or exposure to UVB radiation (80 mJ/cm^2^). Gene expression was normalized to the corresponding untreated control. Data are presented as the mean ± SEM from three independent experiments (n = 3). Statistical significance was evaluated using a paired *t*-test; no significant differences were observed between the mc and mm models.

When normalized to the corresponding untreated control (no IFN-γ stimulation), IFN-γ stimulation did not significantly affect IL-6 expression in either model (ratio ~1) (Figure 4a). In contrast, UVB exposure increased IL-6 expression in both models relative to their corresponding non-irradiated controls, with a greater increase observed in the mm model than in the mc model; however, this difference was not statistically significant.

IDO-1 expression decreased in the mc model after UVB exposure compared to the untreated control (ratio < 1), whereas the mm model showed a marked increase (ratio > 1) (Figure 4b). In contrast to the IL-6 results (Figure 4a), IFN-γ stimulation induced an approximately thousandfold increase in IDO-1 expression in both models, however, with no significant difference between them.

### 3.4. IFN-γ Elevates Kyn Levels in Both Models, While UVB Has a Stronger Effect in the Melanoma Model

**Figure 5:**
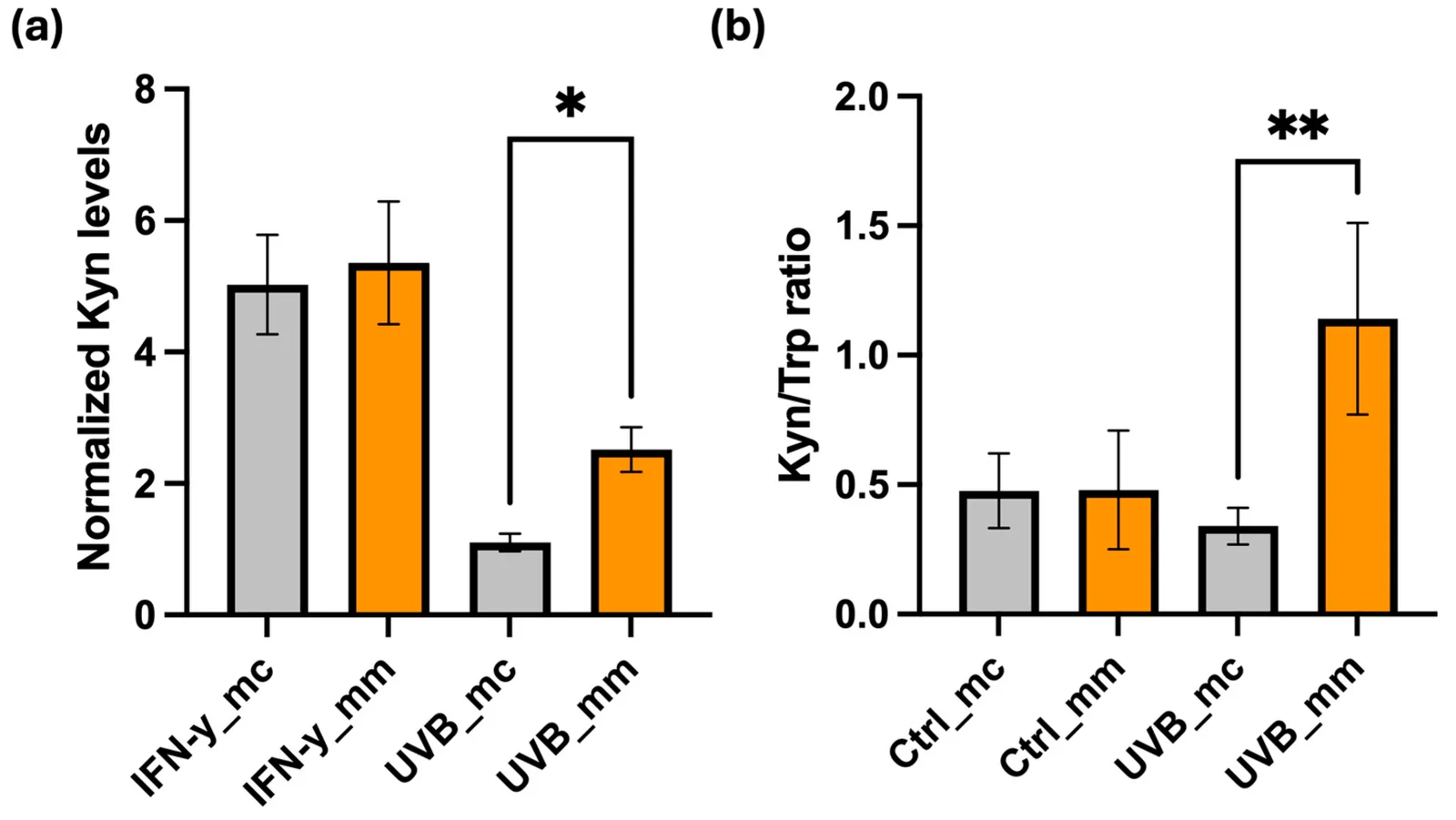
Impact of IFN-γ stimulation or UVB exposure on the normalized basolateral Kyn levels (**a**) and the topical Kyn/Trp ratio (**b**) in mc and mm models. Grey bars represent the mc model, and orange bars represent the mm model. Both models were stimulated with IFN-γ (20 ng/mL) or exposed to UVB radiation (80 mJ/cm^2^). Data are presented as the mean ± SEM from three independent experiments for the mc model and two independent experiments for the mm model. * *p* < 0.05 and ** *p* < 0.01 (paired *t*-test).

Next, the differences in basolateral Kyn levels between both models after IFN-γ or UVB exposure were evaluated using HPLC-PDA analysis. IFN-γ stimulation increased basolateral Kyn concentrations in the mc and mm models, with no significant differences between them (Figure 5a). In contrast, UVB exposure caused a modest increase in basolateral Kyn levels compared to IFN-γ. Kyn levels in the mc model remained near control (ratio ~1), while the mm model showed more than a twofold increase (ratio > 2). In summary, the Kyn concentration was significantly higher in the mm model than in the mc model after UVB exposure (* *p* = 0.0283).

### 3.5. The Topical Ratio of Kyn/Trp in the Melanoma Model Significantly Increases upon UVB Exposure Relative to the Melanocyte Model

Lastly, to evaluate the differences in topical ratios of Kyn/Trp between the two models following UVB exposure, an analysis of Kyn and Trp was performed using HPLC-MS. The effect of UVB on the topical Kyn/Trp ratio varied between both models (Figure 5b), with a notable decrease in the mc model (ratio < 1) and an increase in the mm model (ratio > 1); this difference was statistically significant between the two models (** *p* = 0.0047). IFN-γ stimulation increased topical Kyn/Trp ratios to ~15 in both models, with no significant difference between them.

## 4. Discussion

In this study, two models were developed, one simulating healthy skin (mc model) and another with melanoma cells (mm model), representing disordered skin. While the model developed in this study contains both a dermal and an epidermal compartment arranged in a physiologically relevant 3D configuration, it does not fully replicate the architecture, stratification, or extracellular matrix composition of native human skin. We therefore refer to it as a reconstructed skin equivalent rather than a full-thickness skin model.

The experimental system was based on a 3D approach, in which human skin cells were cultured within a scaffold-supported tissue architecture. This strategy enables improved recapitulation of epidermal–dermal interactions compared with conventional monolayer cultures. Compared with previously reported 3D melanoma and skin models, including spheroids, organoids, and reconstructed skin equivalents, the present system applies a standardized side-by-side experimental design that enables direct comparison between healthy and melanoma-containing skin equivalents within a single scaffold-based platform. This approach aims to minimize variability arising from differences in model composition and experimental setup across studies and supports controlled comparative analysis under identical conditions. 

Skin thickness varies by anatomical site and between individuals, reflecting its role as a biological barrier [22,23]. Additionally, the morphology of the epidermis, characterized by its non-uniform 3D structure with folds and invaginations, contributes to regional differences in skin thickness [24]. The 3D constructs developed distinct epidermal-like and dermal compartments and reproduced selected organizational features observed in native healthy and melanoma skin tissues. Compared with native human skin, the epidermal-like compartment in both models remained thinner and less stratified, with regional variability in thickness. In the mc model, localized downward extensions of epidermal cells into the scaffold were observed. These structures most likely reflect cellular infiltration into scaffold pores, influenced by the structural properties of the scaffold material, rather than genuine rete ridge-like morphogenesis. Although the general morphology superficially resembled rete ridges, we did not observe clear evidence of coordinated dermal–epidermal interdigitation characteristic of native rete ridge formation, and we therefore interpret these structures primarily as scaffold-associated cellular extensions.

In the mm model, the epidermal-like compartment appeared reduced in thickness in regions containing melanoma cell clusters compared with surrounding areas and native melanoma in situ tissue. This observation may reflect local remodeling of the epidermal-like layer associated with melanoma cell growth. Similar epidermal thinning adjacent to melanoma nests has been described in melanoma progression [25]. In addition, compression of surrounding epidermal layers by expanding melanoma nests has previously been reported [26], which may contribute to the morphology observed in the present model.

Melanocytes and melanoma cells in native tissues and 3D skin equivalents were visualized using Melan-A immunostaining, a common clinical marker for detecting benign and malignant melanocytic lesions [27]. However, Melan-A stains all melanocyte-lineage cells [28], limiting its ability to distinguish normal melanocytes from melanoma cells in native skin. Despite this, the reference samples were clinically diagnosed as normal skin and melanoma in situ, and the 3D skin equivalents exhibited similar Melan-A staining patterns. Melanocytes constitute a minority cell population of the epidermis distributed at regular intervals within the stratum basale [29]. In those regions, they form well-organized epidermal-melanin units, each composed of one melanocyte connected to approximately 30–40 adjacent keratinocytes [30], and the Melan-A staining in the mc model is consistent with this arrangement (Figure 2f). During melanoma development, malignant cells grow radially within the epidermis, forming melanoma nests with irregular cell organization and significant variability in cluster size and location [26,31,32]. At this stage, melanoma spreads superficially and remains entirely confined within the epidermis [33,34], hence the term melanoma in situ. Melan-A staining revealed that the melanoma lesions in the mm model resembled those in melanoma in situ, albeit with cell clusters that were less invaginated into the dermis compared to native skin. Several factors could contribute to this, including the rigidity of the polystyrene scaffold limiting cell penetration, as well as the interplay between culture duration and cell division rate, influencing the degree of melanoma cell invagination. Shorter culture duration may limit melanoma cell division and matrix interactions, reducing invagination, whereas longer culture promotes increased cell division and matrix interactions, potentially overcoming scaffold rigidity [35,36]. However, as melanoma cells proliferate in a dense 3D culture, nutrient depletion and spatial constraints may limit the growth of melanoma nests [37,38,39]. Therefore, optimizing these factors could improve the model’s accuracy.

Early detection of skin cancers using biomarkers is essential for increasing patient survival rates. In malignant melanoma, low-molecular-weight biomarkers are particularly valuable because they cross the skin barrier, enabling non-invasive sampling at early disease stages [40,41]. Trp and Kyn are low-molecular-weight molecules whose concentrations and concentration ratios change in response to IDO-1 upregulation. Additionally, both molecules can be topically sampled, making them valuable biomarkers for non-invasive diagnostics [42,43]. Under UVB exposure, the mm model exhibits higher IL-6 (Figure 4a) and IDO-1 (Figure 4b) expression than the mc model. This supports the idea that UVB activates IL-6 and drives IDO-1 upregulation via a positive feedback loop, with a stronger effect in the mm model than in the mc model, suggesting these 3D skin equivalents can reveal specific differences between healthy and diseased states. As a direct metabolite of Trp, Kyn is found at much lower concentrations in tissues and is therefore particularly sensitive to small changes that may indicate significant metabolic alterations associated with melanoma development [42]. A comparative analysis revealed significant differences between the mc and mm models following UVB exposure in basolateral Kyn levels (Figure 5a) and topical Kyn/Trp ratios (Figure 5a), indicating model-specific Kyn pathway responses. These variations may reflect differences in Trp metabolism, tissue damage, or cellular reactions to UVB, underscoring the models’ utility for studying UVB-specific effects and the Kyn/Trp ratio’s value as a skin biomarker of stress and inflammation.

Although UV-induced damage is the primary driver of melanoma development, genetic factors such as mutations in proto-oncogenes or tumor suppressors also play crucial roles, significantly influencing melanoma progression [44]. Among these, the BRAF mutation, found in 50–80% of all melanomas [45,46], is the most common, with the V600E variant accounting for 70–88% of these cases [45]. Interestingly, a recent study demonstrated that BRAF-mutated melanomas exhibit a distinct Kyn pathway gene expression profile compared to wild-type melanomas, suggesting a link between BRAF mutation and the Kyn pathway [47]. The mm model was reconstructed using the Mel-HO cell line, carrying the BRAF V600E mutation, while the mc model incorporated primary melanocytes (HEMa), wild-type for BRAF. The results revealed significant differences in the Kyn pathway between both models, consistent with the findings of Rad Pour et al. [47]. However, using only one melanoma cell line is insufficient to conclude differences in the Kyn pathway profile. Therefore, replacing Mel-Ho with another melanoma cell line in the mm model, e.g., CHL-1 (BRAF wild-type) or Mel-Juso (BRAF wild-type, NRAS mutant), could provide valuable insights into the contribution of distinct driver mutations. In our previous work, we examined the Kyn pathway in monolayers of HEMa and six melanoma cell lines derived from both primary (Mel-HO, Mel-Juso, CHL-1) and metastatic (WM-266-4, SK-MEL-3, HT-144) tumors [18]. These cell lines represent heterogeneous driver mutation backgrounds, including BRAF V600 variants (V600E and V600D) as well as NRAS-mutant and BRAF/NRAS wild-type genotypes. Nevertheless, in our view, melanoma mutation status alone is unlikely to fully explain variability in Kyn pathway expression profiles, underscoring the need for further research.

Moreover, a recent non-invasive pilot study in melanoma patients reported highly variable Trp and Kyn levels in melanoma in situ, with inconclusive results due to the small cohort and confounding factors such as sampling variability, age, and local inflammation, all of which complicate data interpretation in vivo [43]. Nevertheless, our 3D skin equivalents mirror the non-invasive sampling approach used on melanoma patients, reinforcing their value as a controlled systems for investigating Kyn pathway regulation and generating hypotheses for in vivo studies. The mc and mm models were designed as separate, standardized constructs to enable direct comparison of melanocyte and melanoma cell associated responses under identical conditions. The absence of healthy melanocytes in the mm model represents a limitation, as melanocyte–melanoma interactions within the tumor microenvironment may influence proliferation, invasion, and paracrine signaling [14]. Consequently, the IL-6, IDO-1, and Kyn/Trp responses observed in the mm model should be interpreted as responses of a construct containing melanoma cells without concomitant healthy melanocytes and may not fully reflect the biological response of a mixed melanocyte–melanoma microenvironment. The presence of healthy melanocytes could modify the aggregate tissue response, although the direction and magnitude of such effects cannot be determined from the present study. Future studies incorporating keratinocytes, fibroblasts, healthy melanocytes, and melanoma cells within the same reconstructed skin equivalent represent an important direction for further model development. This consideration may be particularly relevant to the Kyn/Trp endpoint, as primary human melanocytes and melanoma cells have previously shown distinct Kyn/Trp responses under inflammatory stimulation [18].

While the mc and mm models have been tested specifically in the context of the Kyn pathway, their potential applications are extensive. Reconstructed 3D skin equivalents, such as those developed here, provide a valuable in vitro platform for investigating UV-related responses. Moreover, they bypass ethical concerns and practical challenges, offering a valuable alternative to human or animal studies. Therefore, these models could be useful for studying the transition of melanocytes into melanoma in situ (melanomagenesis) and its progression to metastatic tumors (metastasis) under repeated UV exposure.

However, the 3D skin equivalents reconstructed in this study have several limitations, including variability in the thickness and organization of the epidermal-like compartment, occasional focal delamination, and the presence of a more compact stratum corneum compared to native skin. The reconstructed skin equivalents were cultured at the ALI, an established approach for promoting keratinocyte stratification, terminal differentiation, and stratum corneum formation [48]. However, formation of a cornified layer in vitro does not necessarily imply complete morphological or functional equivalence to native epidermis. Reconstructed human epidermis has been reported to contain a greater number of compactly packed corneocyte layers than native epidermis, associated with impaired corneocyte desquamation [49]. More recently, human skin equivalent models were shown to develop an excessively thick and compact stratum corneum as a consequence of impaired activation of the desquamation-associated proteases KLK5 and KLK7 and reduced corneodesmosome degradation [50]. These findings provide a plausible explanation for the prominent cornified layer observed in the present models, particularly its compact appearance in the mc model, although desquamation was not directly assessed in our study. The focal delamination observed in some sections may reflect histological-processing artefacts and/or spatial variability in epidermal organization across the construct. Processing-induced separation has been reported in engineered skin equivalents, while inter-batch and spatial variation are recognized considerations in the histological evaluation of reconstructed epidermal models [51,52]. These findings indicate that further optimization of the culture conditions is required. Potential improvements may include adjusting the timing of keratinocyte submersion during 3D culture and modifying the media composition, e.g., excluding antibiotics [53], to promote improved epidermal stratification and tissue organization. 

Although Van Gieson staining demonstrated collagen-containing structures within the dermal compartment (Appendix B), the staining was evaluated qualitatively in the present study, and ECM abundance, organization, and composition were not quantitatively characterized. The presence of fibroblasts and histologically detectable collagen does not, by itself, establish that ECM abundance or organization is comparable to native human dermis, as reconstructed skin equivalents have been reported to exhibit differences in ECM organization and collagen abundance relative to native human skin [10,54]. Future studies should therefore incorporate quantitative and structural ECM analyses, such as biochemical collagen quantification and second-harmonic generation microscopy, to more rigorously characterize dermal matrix maturation.

Another limitation of the present study is that direct viability or cytotoxicity assays were not performed following IFN-γ or UVB stimulation in the reconstructed skin equivalents. Consequently, we cannot fully exclude that some of the observed alterations in Kyn pathway activity were partially influenced by cellular stress or reduced metabolic activity. However, in our previous study using reconstructed human epidermis, IFN-γ stimulation at concentrations up to 50 ng/mL did not reduce tissue viability as assessed by MTT assay, while still inducing measurable inflammatory and Kyn pathway responses [55]. Based on these findings, a lower IFN-γ concentration (20 ng/mL) was selected in the present study to induce biological pathway activation while minimizing the risk of viability-related effects. In contrast, the effects of UVB exposure at 80 mJ/cm^2^ on tissue viability were not directly evaluated in the current 3D skin equivalents, and UVB-induced cytotoxic effects therefore cannot be fully excluded. Nevertheless, comparable UVB doses have previously been used in reconstructed skin models to induce measurable inflammatory and stress-associated responses without immediate tissue destruction or complete loss of viability [56,57]. In the present study, histological evaluation following UVB exposure indicated epidermal morphological alterations, whereas the dermal compartment and collagen organization appeared largely unchanged. However, these observations do not provide direct information regarding tissue viability or functional barrier properties. Furthermore, the heterogeneous epidermal thickness and limited barrier function of reconstructed tissues may influence UVB penetration and cellular sensitivity compared with native human skin. Future studies should therefore combine metabolic analyses with dedicated viability, apoptosis, and barrier integrity assays to further validate the physiological responses of the model.

A key step in advancing both models is incorporating immune cells, such as Langerhans cells, macrophages, or dendritic cells, to enable a more comprehensive study of the skin’s immune response to UV light and its role in skin health and disease. Simultaneously, incorporating functional vasculature with capillary-like structures is essential to enhance physiological relevance, better replicate in vivo skin circulation, and improve the models’ viability and barrier function [58]. 

Finally, although reconstructed skin equivalents are valuable, they do not provide a universal solution, but are designed to address specific research questions. Therefore, their true value lies in serving as in vitro tools to bridge knowledge gaps in specific areas of skin research. To effectively utilize these models, it is essential to consider the biological context and limitations of each model application. Within these limitations, 3D skin equivalents may complement traditional experimental systems by enabling investigation of selected aspects of skin physiology and pathology in a controlled in vitro setting.

## 5. Conclusions

The reconstructed skin equivalents developed epidermal-like and dermal compartments incorporating either melanocytic or melanoma cell populations, enabling investigation of selected structural and functional features associated with healthy and diseased skin. UVB exposure induced epidermal morphological changes, including altered keratinocyte organization and changes in the superficial cornified layer, while the overall dermal compartment and collagen organization appeared preserved. In addition, UVB exposure revealed differences in Kyn pathway activity between the models, indicating melanoma-associated alterations in pathway regulation. Although the skin equivalents did not fully recapitulate native human skin morphology or architecture, these comparative 3D culture systems provide useful in vitro platforms for studying defined aspects of skin biology and melanoma-related processes in a controlled experimental environment.

## Figures and Tables

**Table 1:** Culture media composition for fibroblasts, melanocytes, melanoma cells, and keratinocytes.

Cell Type	Basal Medium	Supplements	Antibiotics
Fibroblasts (BJ)	EMEM	10% FBS	1% PEST
Melanocytes (HEMa)	Dermal Cell Basal Medium	Adult Melanocyte Growth Kit	1% PEST
Melanoma cells (Mel-HO)	DMEM with 1 g/L glucose	10% FBS	1% PEST
Keratinocytes (HEKn)	EpiLife with 60 µM calcium	1% HKGS	0.2% gentamicin sulfate

EMEM, Eagle’s Minimum Essential Medium; DMEM, Dulbecco’s Modified Eagle Medium; FBS, fetal bovine serum; PEST, penicillin/streptomycin; HKGS, Human Keratinocyte Growth Supplement.

**Table 2:** Composition of the mc and mm model co-culture growth media.

Medium Fraction	Composition
Shared basal fraction (80%)	EpiLife (60 µM calcium) supplemented with 1% HKGS and 0.2% gentamicin sulfate
mc-specific fraction (20%)	Dermal Cell Basal Medium supplemented with Adult Melanocyte Growth Kit and 1% PEST
mm-specific fraction (20%)	DMEM (1 g/L glucose) supplemented with 10% FBS and 1% PEST
Fresh supplementation	50 µg/mL L-ascorbic acid 2-phosphate

**Table 3:** Composition of the mc and mm model differentiation media.

Medium Fraction	Composition
Shared basal fraction (80%)	EpiLife (60 µM calcium) supplemented with 1% HKGS and 0.2% gentamicin sulfate
mc-specific fraction (20%)	Dermal Cell Basal Medium supplemented with Adult Melanocyte Growth Kit and 1% PEST
mm-specific fraction (20%)	DMEM (1 g/L glucose) supplemented with 10% FBS and 1% PEST
Fresh supplementation	50 µg/mL L-ascorbic acid 2-phosphate, 1.5 mM calcium chloride, and 10 ng/mL recombinant human keratinocyte growth factor

## Data Availability

The raw data that support the findings of this study are available upon request from the corresponding author.

## Abbreviations

The following abbreviations are used in this manuscript:

3D	three-dimensional
ALI	air-liquid interface
DMEM	Dulbecco’s Modified Eagle Medium
ECM	extracellular matrix
EMEM	Eagle’s Minimum Essential Medium
FBS	fetal bovine serum
HC	histochemical staining
HEMa	human epidermal melanocytes, adult
HEKn	human epidermal keratinocytes, neonatal
H&E	hematoxylin and eosin
HKGS	human keratinocyte growth supplement
HPLC-MS	high-performance liquid chromatography–mass spectrometry
HPLC-PDA	high-performance liquid chromatography–photodiode array detector
IDO-1	indoleamine 2,3-dioxygenase 1
IFN-γ	interferon-gamma
IHC	immunohistochemical staining
IL-6	interleukin-6
Kyn	kynurenine
mc	melanocyte model
mm	melanoma model
NAD+	nicotinamide adenine dinucleotide
PEST	penicillin–streptomycin
RT	room temperature
RT-qPCR	reverse transcriptase quantitative polymerase chain reaction
Trp	tryptophan
UV	ultraviolet

## Appendix A

### Appendix A.1. Downstream Analysis Procedures

The following methods were used for downstream analyses of the reconstructed skin equivalents. Due to differences in analyte concentration and sample composition between basolateral and topical compartments, different analytical approaches were applied for Kyn and Trp quantification. Kyn levels in basolateral samples were quantified using HPLC-PDA, whereas the more sensitive HPLC-MS method was used for quantification of Trp and Kyn in topical samples.

### Appendix A.1.1. HPLC-PDA Analysis

Prior to Kyn analysis, proteins were removed from basolateral samples using 10 kDa molecular weight cut-off filters (PES modified; VWR International; Radnor, PA, USA). Samples (up to 500 µL) were centrifuged at 10,000–14,000× *g* at 4 °C for 15 min or until complete filtration. Kyn quantification was performed using an Agilent 1100 Series HPLC system equipped with a PDA detector (Agilent Technologies; Waldbronn, Germany). Chromatographic separation was achieved on a reversed-phase Kromasil C18 column (250 × 4.6 mm, 5 μm particle size, 100 Å; ES Industries; West Berlin, NJ, USA) using a gradient of 10 mM NaH_2_PO_4_ (pH 2.8) and methanol at a flow rate of 0.9 mL/min and a column temperature of 40 °C. Kyn was detected at 360 nm. Quantification was based on a calibration curve ranging from 0.78 to 100 μM (R^2^ = 0.99). The limit of detection (LOD) and the limit of quantification (LOQ) values ranged from 0.2–0.4 μM and 0.5–0.7 μM, respectively. These slight variations in LODs and LOQs between the mc and mm models are attributed to differences in the cell culture media used for growing these models. Samples and standards were analyzed in triplicate. Detailed validation parameters, including precision and accuracy, were previously described by Hasterok et al. [42].

#### Appendix A.1.2. HPLC-MS Analysis

Topical (PBS-based) samples were analyzed without prior filtration. Quantification of Trp and Kyn was performed using an HPLC system (Alliance 2695; Waters, Milford, MA, USA) coupled to a Micromass ZQ mass spectrometer with electrospray ionization (ESI). Chromatographic separation was achieved on a Kromasil C18 reversed-phase column (250 × 4.6 mm, 5 µm particle size, 100 Å; ES Industries) using gradient elution with 0.1% formic acid in water and methanol at a flow rate of 0.8 mL/min. Quantification was carried out in selected ion recording (SIR) mode with positive ionization. Calibration curves ranged from 0.15 to 5 µM for both Trp and Kyn (R^2^ > 0.99). LODs were 0.1 µM for Trp and 0.04 µM for Kyn, while LOQs were 0.3 µM and 0.1 µM, respectively. Detailed information regarding method validation, including precision and accuracy, has been previously described by Hasterok et al. [42].

#### Appendix A.1.3. Histological and Immunohistochemical Staining of Reconstructed Skin Equivalents and Human Tissue Sections

Following fixation in formaldehyde for 24 h, the reconstructed skin equivalents were excised from inserts, dehydrated, paraffin-embedded, and sectioned at 3 μm thickness. Commercial formalin-fixed paraffin-embedded (FFPE) human tissue sections containing melanoma in situ (CS807094, OriGene, Rockville, MD, USA) and normal skin (CS703949, OriGene, Rockville, MD, USA) were used for comparison and sectioned at 5 μm thickness.

Tissue sections from reconstructed skin equivalents and native human tissues were stained with hematoxylin and eosin (H&E) using standard protocols or subjected to histological and immunohistochemical analyses.

For collagen visualization, sections were stained using Van Gieson’s method according to standard protocols. Briefly, deparaffinized and rehydrated sections were stained with Weigert’s iron hematoxylin, followed by counterstaining with Van Gieson solution to visualize collagen fibers. For immunohistochemical staining of melanocytic and epithelial markers, sections were deparaffinized, rehydrated, and subjected to heat-induced antigen retrieval in citrate buffer (10 mM, pH 6.0) at 95 °C for 40 min. After blocking of non-specific staining (MACH4 kit, BRI4012L; Biocare Medical, Pacheco, CA, USA), sections were incubated overnight with monoclonal mouse anti-human Melan-A antibody (Clone A103, DAKO; 1:400 dilution) or monoclonal mouse anti-human cytokeratin AE1/AE3 antibody (M3515, DAKO; dilution 1:100). Immunodetection was performed using the MACH4 detection system with DAB visualization (Biocare Medical), followed by hematoxylin counterstaining. Tonsil tissue and mouse negative control reagent (N1698; DAKO) were used as positive and negative controls, respectively.

#### Appendix A.1.4. RNA Isolation and RT-qPCR

Total RNA was isolated from whole mc and mm models using the RNeasy Mini Kit (Qiagen; Hilden, Germany) according to the manufacturer’s instructions, including β-mercaptoethanol-assisted lysis and ethanol-based RNA purification. Reverse transcription was performed using the SensiFAST cDNA Synthesis Kit (Bioline; London, UK). Quantitative PCR (qPCR) was carried out using SYBR Green chemistry (Bioline) on a LightCycler 480 instrument (Roche; Basel, Switzerland). Amplification conditions consisted of an initial denaturation at 95 °C for 10 min, followed by 45 cycles of 95 °C for 10 s, 65 °C for 10 s, and 72 °C for 11 s. GAPDH was used as the reference gene. Primer sequences and corresponding NCBI gene IDs are listed in Table A1. Relative gene expression was calculated using the ΔΔCt method [59], with unstimulated mc and mm models used as calibrators. The baseline gene expression from unstimulated models is represented as “1”.

**Table A1:** NCBI gene IDs and primer sequences of human genes targeted in RT-qPCR.

Glyceraldehyde-3-phosphate dehydrogenase (GAPDH)	
NCBI gene ID:	2597
Forward primer:	AACAGCGACACCCACTCCTC
Reverse primer:	GGAGGGGAGATTCAGTGTGGT
Interleukin 6 (IL-6)	
NCBI gene ID:	3569
Forward primer:	AGACAGCCACTCACCTCTTCAG
Reverse primer:	TTCTGCCAGTGCCTCTTTGCTG
Indoleamine 2,3-dioxygenase 1 (IDO-1)	
NCBI gene ID:	3620
Forward primer:	GAAAGGCAACCCCCAGCTAT
Reverse primer:	GGAGGAACTGAGCAGCATGT

## Appendix B

### Additional Histological Characterization of Reconstructed Skin Equivalents

Representative AE1/AE3 immunohistochemical and Van Gieson staining were performed to further characterize the reconstructed mc and mm models under control and UVB-treated conditions. AE1/AE3 staining highlighted the reconstructed epidermal compartment in both models, whereas Van Gieson staining confirmed the presence and distribution of collagen fibers within the reconstructed dermal compartment (Figure A1 and Figure A2). Following UVB exposure, the reconstructed models exhibited morphological changes in the epidermal compartment, including a less regularly organized appearance of basal keratinocytes and an apparent increase in stratum corneum thickness. The stratum corneum appeared less compact and more loosely organized compared with the corresponding controls. No apparent changes in collagen fiber organization were observed following UVB exposure. Despite these epidermal alterations, the epidermal and dermal compartments remained distinguishable in both reconstructed skin equivalents.
